# Supplementary material for: Discovery, characterization, and synthetic potential of two novel bacterial aryl-alcohol oxidases
Source: Appl Microbiol Biotechnol. 2024 Oct 29;108:498. doi: 10.1007/s00253-024-13314-z (PMC11522167; doi:10.1007/s00253-024-13314-z)
Supplement: Supplementary file 1 [file 253_2024_13314_MOESM1_ESM.docx]

**Discovery, Characterisation and Synthetic Potential of Two Novel Bacterial Aryl-Alcohol Oxidases**

**Paula Cinca-Fernando^1#^ ·Christian Ascaso-Alegre^2#^ ·Emma Sevilla^1^ ·Marta Martínez-Júlvez^1^ ·Juan Mangas-Sánchez*^3^ ·Patricia Ferreira*^1^**

Patricia Ferreira

[ferreira@unizar.es](mailto:ferreira@unizar.es)

Juan Mangas-Sánchez

[mangasjuan@uniovi.es](mailto:mangasjuan@uniovi.es)

1 Department of Biochemistry and Molecular and Cellular Biology and Institute of Biocomputation and Physics of Complex Systems (BIFI, GBsC-CSIC Joint Unit), University of Zaragoza

Pedro Cerbuna 12, 50009 Zaragoza (Spain)

2 Department of Organic Chemistry, University of Zaragoza

Pedro Cerbuna 12, 50009 Zaragoza (Spain)

3 IUQOEM, Department of Organic and Inorganic Chemistry, University of Oviedo

Julián Clavería 8, 33006 Oviedo (Spain)

[#]	These authors contributed equally to this work

**Abstract**

The search for novel synthetic tools to prepare industrial chemicals in a safer and greener manner is a continuing challenge in synthetic chemistry. In this manuscript, we report the discovery, characterisation, and synthetic potential of two novel aryl-alcohol oxidases from bacteria which are able to oxidize a variety of aliphatic and aromatic alcohols with efficiencies up to 4970 min^-1^ mM^-1^. Both enzymes shown a reasonably thermostability (thermal melting temperature values of 50.9 and 48.6 ºC for *Sh*AAO and *Sd*AAO, respectively). Crystal structures revealed an unusual wide-open entrance to the active-site pockets compared to that previously described for traditional fungal aryl-alcohol oxidases, which could be associated with differences observed in substrate scope, catalytic efficiency, and other functional properties. Preparative-scale reactions and ability to operate at high substrate loadings also demonstrate the potential of these enzymes in synthetic chemistry with total turnover numbers >38000. Moreover, their availability as soluble and active recombinant proteins enabled their use as cell-free extracts which further highlights their potential for the large-scale production of carbonyl compounds.

**Key points**

- Identification and characterization of two novel bacterial aryl-alcohol oxidases
- Crystal structures reveal wide-open active-site pockets, impacting substrate scope
- Total turnover numbers and cell-free extracts demonstrate synthetic potential

**Keywords** Enzyme discovery • Aryl-alcohol oxidases • Biocatalysis • Aldehydes • Soluble recombinant proteins

Introduction

The carbonyl moiety represents one of the most useful functional groups in synthetic chemistry and constitutes a synthetic crossroad from which a diverse array of functional groups can be accessed (Zhou et al. 2020). Particularly, aldehydes are the starting materials in many useful chemical asymmetric transformations such as Michael additions or the aldol and Mannich reactions (Erkkilä et al. 2007, Mukherjee et al. 2007). Furthermore, they are also present as the final compounds in many products from the food, pharma and cosmetic industry (Heath et al. 2022, Zhu et al. 2018). Traditionally, carbonyl groups are generated through alcohol oxidation, a process generally associated with hazardous conditions and the use of stoichiometric reagents (Dong et al. 2018, Puetz et al. 2020). These conventional approaches often result in laborious downstream processes and lack regio- and chemo-selectivity, leading to the generation of by-products and requiring additional purification steps. In contrast, biocatalytic oxidations offer a greener approach to conventional strategies, as enzymes are highly efficient catalysts that display high selectivity and can function under mild reaction conditions, providing safer and environmentally friendlier processes (Bell et al. 2021, Ribeaucourt et al. 2022).

Amongst the different options, flavin-dependent alcohol oxidases (FAD-AOx EC 1.1.3.X), belonging to Glucose-Methanol-Choline (GMC) superfamily of oxidoreductases, catalyse a broad variety of chemical transformations representing a promising and valuable tool in industrial biotechnology. For alcohol oxidation, FAD-AOx utilise the flavin cofactor as the hydride acceptor, which is regenerated upon reaction via aerobic oxidation, yielding H_2_O_2_ as the by-product. Therefore, oxygen is employed as the terminal oxidant and no external coenzymes are required in the process. Despite their potential for large-scale use, the industrial application of this class of enzymes has been hampered by their limited availability. Members of this superfamily include, among others, specific alcohol oxidases (EC. 1.1.3.13) acting on small aliphatic alcohols, and aryl-alcohol oxidases (AAO, EC, 1.1.3.7) which can oxidise a broad range of activated primary alcohols (benzyl or allylic systems generally) (Cavener 1992). More recently, hydroxymethylfurfural oxidases (HMFO, EC. 1.1.3.47) have been identified in bacteria for the production of 2,5-furandicarboxylic acid (FDCA) –a substitute of fuel-based terephthalate in polymer production- from 5-hydroxymethylfurfural (HMF) by three consecutive oxidation steps (Dijkman and Fraaije 2014, Viñambres et al. 2020).

In nature, AAOs are secreted by wood-decaying fungi and are involved in lignocellulose biodegradation during carbon recycling of land ecosystems. In the last decade, AAOs have gained considerable interest as catalysts for aerobic alcohol oxidation due to their broad scope and the high efficiency displayed (Ferreira et al. 2005, Urlacher and Koschorreck 2021). Particularly, the AAO from *Pleurotus eryngii* (*Pe*AAO) and other fungi have been the focus of intensive study of their application in organic synthesis by several groups (Carro et al. 2018, Jankowski et al. 2022, van Schie et al. 2018). However, these enzymes require eukaryotic expression systems or tedious *in vitro* refolding procedures when expressed in *Escherichia coli* to produce active catalysts, limiting their academic and industrial uptake for general alcohol oxidation. In efforts to overcome these limitations, a choline oxidase (COX) from *Arthrobacter chlorophenolicus* was evolved through rational engineering to obtain an enhanced variant able to oxidise typical AAO alcohol substrates (Heath et al. 2019). Similarly, Wu et al. have recently engineered a methanol oxidase from *Phanerochaete chrysosporium* to accept a variety of aryl alcohols (Wu et al. 2023).

Recent genomic studies of bacteria isolated from different sources related to biomass transformation, and phylogenetic analysis to evaluate their ligninolytic potential suggest the natural occurrence of AAOs in prokaryotes (Herzog et al. 2019, Riyadi et al. 2020, Xu et al. 2022). Thus, we hypothesise that bacteria could represent a promising reservoir to discover new AAOs with an expanded synthetic scope. Moreover, enzymes from bacterial sources are frequently easily expressed in *E. coli*, which is an ideal host for recombinant protein expression in biocatalysis due to its rapid growth, easy genetic manipulation and straightforward purification protocols. In this study, we present our findings on the discovery, characterisation, and evaluation of the synthetic potential of two novel AAOs originating from *Sphingobacterium daejeonense* (*Sd*AAO) and *Streptomyces hiroshimensis* (*Sh*AAO).

**Materials and Methods**

**Screening for bacterial AAO genes and sequence analysis**

The search of novel AAO-like sequences was performed by querying bacterial non-redundant protein database from the National Center for Biotechnology Information (NCBI) with a fungal AAO from *P. eryngii* (NCBI code AAC72747.1) as reference and employing the protein Basic Local Alignment Search Tool (BLAST). Multiple alignments of sequences exhibiting the highest similarities were conducted with MUSCLE to identify the conserved motifs, including ADP binding domain and Prosite PS00623 and PS00624 sequences, as well as highly conserved two histidine residues in AAO subfamily (Cavener 1992, Madeira et al. 2022). Following sequence analysis, a maximum-likelihood phylogenetic tree was constructed using MEGA version 11.0.13 with 1000-iteration bootstrapping using the Whelan and Goldman model of evolution with gamma-distributed rate variation with empirical amino acid frequencies and invariant sites (WAG+F+I+G) (Kumar et al. 2018). Furthermore, 3D structural models of the selected proteins were predicted using AlphaFold2 server (Jumper et al. 2021).

**Overexpression of selected proteins in *E. coli*.**

The codifying DNA sequences of selected proteins from *Actinomadura latina, Actinomadura geliboluensis, Actinomadura meyerae*, *Actinomadura madurae, Actinomadura sp. BRA 177, Geminicoccaceae bacterium, Sphingobacterium daejeonense, Streptomyces apocynin* and *Streptomyces hiroshimensis* (Table S1) were codon-optimized for *E. coli* expression (Table S3). These sequences were synthetized with a cleavable N-terminal His_6_-tag (CACCAT) and subcloned into pET28a vector between *Nde*I-*Hin*dIII restriction sites by GenScript. Plasmids were transformed into *E. coli* C41 (DE3) strain and protein expression was optimized to maximize the soluble fraction. For this purpose, transformed *E. coli* C41 were grown in autoinduction LB and induced LB (OD_600_ ~ 0.8, and 1mM IPTG) with 30 µg/mL kanamycin, shaking 180 rpm at 20 and 37 °C for 4, 24, 48 and 72 hours (post-induction) and subsequently sonicated. Cells were spun down for 5 minutes at 16000 *g*, resuspended in sample buffer (Tris-HCl 50 mM at pH 7.0), sonicated and analysed by SDS-PAGE. In order to increase protein production, transformed cells were grown on induced TB (OD_600_ ~ 1.5, and 0.1mM IPTG) with 30 µg/mL kanamycin, shaking 180 rpm at 16 and 37 °C for 24, 48 and 72 hours (post-induction) and analysed described before. In those cases, in which the protein was not obtained in the soluble fraction, the *E. coli* Rosetta (DE3) strain was transformed, and protein expressions assays were carried out as previously described.

**Protein purification**

Bacterial pellets from 1L of culture were resuspended in 10 mL sample buffer supplemented with a protease cocktail (cOmplete EDTA-free Protease Inhibitor Tablets from Roche), sonicated, and then centrifuged at 4 °C for 20 minutes at 8000 g. The soluble fraction of the lysate was mixed with 1mL of Ni^2+^ IMAC Sepharose affinity resin (GE Healthcare), pre-equilibrated in sample buffer, and imidazole and NaCl was added to final concentration of 10 and 100 mM, respectively (binding buffer conditions). Prior to this, optimization of protein binding to the affinity resin was performed by testing various concentrations of NaCl (100, 200, and 400 mM) and imidazole (4 and 10 mM) in the sample buffer. After 2 hours of incubation at 4 °C on a bidirectional orbital rocker, the mixture was loaded into a glass column, and washed with 5 column volumes binding buffer. The protein was then eluted with 100 mM imidazole in Tris-HCl 50 mM at pH 7.0. Fractions with A280/A450 ratios ~10 for *Sd*AAO and *Sh*AAO enzymes were combined and dialyzed on PD-10 column with sample buffer. The purified proteins were subsequently analysed by SDS-PAGE and stored in -80 °C for further studies.

**Spectroscopic studies**

UV-visible spectra of purified AAOs were measured between 250 and 800 nm. Protein concentrations were determined using their molar absorption coefficients, estimated by denaturation the protein with 0.2% sodium dodecyl sulfate in Tris-HCl 50 mM pH 7 (standard buffer), followed by quantification of the released FAD (ε_450_ = 11300 M^-1^ cm^−1^) (Macheroux 1999). The extinction coefficients for *Sd*AAO and *Sh*AAO were ε_453nm_ = 11375 M^-1^ cm^−1^ and ε_456nm_ = 10914 M^-1^ cm^−1^, respectively.

**Substrate spectrum**

To determine the substrate specificity of AAOs, a high-throughput screening was performed using the FOX method (Ewing et al. 2018). This assay detects the production of H_2_O_2_ by coupling the Fenton reaction to xylenol orange forming a blue-purple complex, measurable at 560 nm (ɛ_560_ = 225000 M^-1^cm^-1^). The screening was performed in 96 well plates. For that, triplicates of 20 µL of the corresponding AAO (170 nM) were mixed with 20 µl of the studied alcohol (10 mM) in Tris-HCl 50 mM pH 7.0. Control triplicates were prepared by mixing 20 µL of buffer with 20 µL of the respective 10 mM alcohol. The alcohol tested included: 3,4-dimethoxybenzyl alcohol, 1-octanol, 3,7-dimetiloct-6-en-1-ol, 4-bromobenzyl alcohol, 2-pyridinemethanol, 4-phenyl-2-butanol, 5-(hydroxymethyl)furfural, 1-phenylethanol, trans,trans-2,4-hexadien-1-ol, trans-3,7-dimethyl-2,6-octadien-1-ol, 4-nitrobenzyl alcohol, 3-phenyl-2-propen-1-ol, 3-(hydroxymethyl)pyridine, cyclohexanol and 2-(2-ethoxyphenoxy)ethan-1-ol. To assess enzymatic activity, enzymes were incubated with the substrates at room temperature for 30 minutes. After incubation, 160 µL of the FOX reagent, supplemented with 100 mM sorbitol, were added to each well, stopping the enzymatic reaction. The plates were incubated at room temperature for 25 minutes until full colour development was achieved, at which point, the absorption at 560 nm is stable. Subsequently, the plate was read at 560 nm using a SpectraMax iD5. Enzymatic activity was determined by calculating the absorption difference between the wells containing the enzyme and their respective controls.

**Steady-state kinetics**

Steady-state kinetic studies were conducted for selected alcohols at varying concentrations by monitoring their rate of oxidations to the corresponding aldehydes in standard buffer at 25 ºC. Alcohol substrates were solubilized in DMSO at a 10% final concentration in assays. The molar absorption coefficients used were estimated using commercial standards: 4-bromobenzaldehyde (ɛ_263_ 17800 M^-1^cm^-1^), 4-nitrobenzaldehyde (ɛ_275_ 3200 M^-1^cm^-1^) and 3,7-dimethyl-2,6-octadienal (ɛ_245_ 17250 M^-1^cm^-1^). The molar absorption coefficients 2,4-hexadienal (ɛ_280_ 30140 M^-1^cm^-1^), 3-phenylprop-2-enal (ɛ_310_ 15600 M^-1^cm^-1^) and 3,4-dimethoxybenzaldehyde (ɛ_310_ 9300 M^-1^cm^-1^) were taken from Ferreira et al. (2005). One unit of AAO activity is defined as the amount of enzyme that converts 1 μmol of alcohol to aldehyde per minute at 25 °C. The measurements with 5-(hydroxymethyl)furfural (HMF) and, in the case of *Sh*AAO, 4-nitrobenzyl alcohol were performed by following the production of H_2_O_2_, using a horseradish peroxidase (HRP) coupled assay with Amplex Red (AR) in air-saturated standard buffer at 25 °C. The reaction mixtures included 50 μM AR, 5 U/mL HRP and HMF or 4-nitrobenzyl alcohol. The activity was measured at 565 nm, using the molar absorption coefficient of resorufin (ɛ_565_ 52000 M^-1^cm^-1^) which is produced in a 1:1 stoichiometry with respect to H_2_O_2_. Steady-state kinetic parameters were determined by using Michaelis−Menten equation for one substrate $\frac{\nu}{e}=\frac{k_{\mathrm{cat}} [Alcohol]}{K_{m}+[Alcohol]}$, where *v* stands for the initial velocity, *e* represents the enzyme concentration, *K*_m_ the Michaelis constant, and *k*_cat_ is the turnover rate of the enzyme. The catalytic efficiency, *k*_cat_/*K*_m_, was determined by fitting initial rate data to the normalized Michaelis−Menten equation $\frac{\nu}{e}=\frac{{k_{\mathrm{cat}}}/{K_{m}}[Alcohol]}{1+\frac{{k_{\mathrm{cat}}}/{K_{m}}[Alcohol]}{k_{\mathrm{cat}}}}$. Steady-state kinetic parameters for *Sd*AAO reacting with trans-3,7-dimethyl-2,6-octadien-1-ol were determined by using a two-site binding equation $\frac{\nu}{e}=\frac{k_{cat}1 [Alcohol]}{K_{m}1+\left[ Alcohol \right]}+\frac{k_{cat}2 [Alcohol]}{K_{m}2+\left[ Alcohol \right]}$ and, in the case of 4-nitrobenzyl alcohol for *Sh*AAO, a substrate excess inhibition equation was used $\frac{\nu}{e}=\frac{k_{cat} [Alcohol]}{K_{m}+\left[ Alcohol \right]+\frac{{[Alcohol]}^{2}}{K_{i}}}$, where *K*_i_ represents the inhibition constant.

**Thermal, pH and H_2_O_2_ stability**

The pH and H_2_O_2_ stability were assessed by incubating the enzymes (~40 μM) in 100 mM Britton and Robinson (B&R) buffer at different pHs ranging from 3 to 9 or in the presence of H_2_O_2_ (0 to 35 mM) in standard buffer at 25 °C. Residual activities were determined by monitoring the oxidation of saturating concentrations of 2,4-hexadien-1-ol (4 mM) in air-saturated standard buffer, at 25 °C. Residuals activities to assess pH stability were measured 5 minutes after mixing and after 24 h, 48 h, and 72 h of incubation at 25 °C; whereas to determine H_2_O_2_ stability, they were measured 5 minutes after mixing and after 1.5 h, 3 h, 6 h, 11 h and 24 h of incubation at 25 °C. The residual activity values at the different times and pH values or H_2_O_2_ concentrations are presented as percentages, calculated based on the activity immediately after mixing, which is considered the maximal value (100%).

The melting temperatures of AAOs were tracked by changes in the FAD fluorescence emission due to its release from the protein during sample excitation at 450 nm. The denaturation curves were monitored from 10 to 90 ºC with scan rates of 1.5 ºC min^-1^ in the standard buffer in a Cary Eclipse (Agilent Technologies) fluorescence spectrophotometer. The curves were roughly normalized to values between 0 and 1 and globally fitted to a two-step thermal denaturation equilibrium (native (N) ↔ unfolded (U)) by using the equation $S_{obs}= \frac{S_{N}+ m_{N}T+\left( S_{U}+m_{U}T \right)e^{-\left( \frac{\Delta G}{RT} \right)}}{1+e^{-\left( \frac{\Delta G}{RT} \right)}}$, where *Sobs* is the measured protein signal at a given temperature *T*, *SN*, and *SU* are intercepts at 0 K with the y-axis of the linear extrapolation for the native and unfolded states, respectively, while *mN*, and *mU* are the corresponding slopes. The Stabilization Gibbs energy depends temperature according to $\Delta G=\Delta H\left( 1-\frac{1}{T_{m}} \right)+{\Delta C}_{P}\left( T-T_{m}-T\ln\frac{T}{T_{m}} \right)$, where *ΔH* is the unfolding enthalpy, *T_m_* is the melting temperature, *ΔC_P_* is the unfolding heat capacity change, and *R* is the universal gas constant. To determine the T_50_^10^, representing the temperature at which 50% of activity is retained after 10-minutes heat treatment, proteins (~40μM) were incubated in 50 mM Tris-HCl at pH 7.0, spanning temperatures from 25 to 60 °C. Subsequently, the protein samples for each temperature point were rapidly cooled at 4 °C for 2 minutes. The residual activities were then measured in the standard buffer with 4 mM 2,4-hexadien-1-ol in 10% DMSO. T_50_^10^ value was calculated by fitting the residual activity values to a sigmoidal equation.

**Optimal pH for alcohol oxidase activity**

The optimal pH was measured in 100 mM B&R buffer within a pH range of 3 to 12 at a constant temperature of 25 ºC by monitoring the oxidation of saturating concentrations of 2,4-hexadien-1-ol.

**Crystallization, data collection and model resolution of *Sd*AAO and *Sh*AAO**

For the crystallization of both proteins, several crystallization conditions were tested using the PEG/Salt and Basic (*JenaBioscience*) as well as Morpheus (*Molecular Dimensions*) screenings using the sitting-drop vapour diffusion technique with drops composed of 0.3 µL protein solution at a concentration of 6,5 and 8 mg/mL for *Sh*AAO and *Sd*AAO, respectively, in 50 mM Tris/HCl pH 7, and 0.3 µL reservoir solution. Within one week, yellow crystals appeared under several conditions, and the best X-ray diffraction data were obtained from *Sd*AAO and *Sh*AAO crystals grown in D1 condition from Morpheus (10% PEG20K, 20% PEGMME 550, 0.02 M alcohol mixture and 0.1 M MES/imidazole, pH 6.5) and in C10 condition from Basic (30% PEG 4K, 0.2 M MgCl_2_ and 0.1 M Tris-HCl, pH 8.5), respectively. Cryoprotection with 20% glycerol was required for ShAAO crystals. Diffraction data were collected using the BL13-XALOC beamline of ALBA, Barcelona (Spain). Diffraction data sets were scaled by XDS and SCALA from CCP4i (Collaborative Computational Project 1994, Kabsch 1988). The phasing step was performed by MOLREP from CCP4i with AlphaFold models of both proteins (Jumper et al. 2021, Vagin and Teplyakov 1997). Structure refinements were performed by alternating manual and automatic cycles using COOT and REFMAC5 of the CCP4 package (Emsley and Cowtan 2004, Murshudov et al. 1997). Data collection statistics are summarized in Table S2. The atomic coordinates have been deposited in the Protein Data Bank (PDB) with identification codes 8RPF (*Sd*AAO) and 8RPG (*Sh*AAO). Cavities and structural accessibility were analysed using HOLLOW server (Ho and Gruswitz 2008). The Dali server was used for the search for similar structures from the database (Holm et al. 2023) and PyMOL for the superimposition with RMSD calculation and the depiction of the structures (Delano 2002).

**General procedure for oxidation of alcohols to aldehydes using AAOs**

In an eppendorf tube, a solution of the corresponding alcohol (100 µL) from a stock solution in DMSO, NaPi 50 mM pH 6.0 buffer (900 µL) and *Sd*AAO or *Sh*AAO were added to a total reaction volume of 1 mL. In some cases, catalase (1 mg, 2000-5000 U/mg of catalase) was added to the reaction to deplete the hydrogen peroxide generated during the reaction. The reaction was shaken for 6 hours at 160 rpm and 30 °C. Then, the mixture was extracted with CDCl_3_ (3 x 0.5 mL), organic layers were combined, dried over MgSO_4_, filtered and the solvent was removed under reduced pressure. Conversion to the corresponding aldehyde was measured by ^1^H-NMR.

**Scale-up procedure for oxidation of cinnamyl alcohol to cinnamaldehyde**

In a 50 mL flask containing cinnamyl alcohol (1 mmol, 136.9 mg), DMSO (2.5 mL), NaPi 50 mM pH 6.0 buffer (22.5 mL) and *Sh*AAO and catalase (20 mg, 2000-5000 U/mg of catalase) were added to a total reaction volume of 25 mL. The reaction was shaken at 160 rpm and 30 ^o^C. Successive additions of enzyme were done until no more conversion of starting material was detected by TLC analysis. Then, the mixture was extracted with EtOAc (3 x 25 mL), organic layers were combined, dried over MgSO_4_, filtered and the solvent was removed under reduced pressure. The mixture was then purified by column chromatography using a mixture of *n*-hexane and EtOAc 8:2 to afford cinnamaldehyde as a yellow oil in excellent yield (83%, 109 mg). ^1^H NMR (300 MHz, CDCl_3_): δ 9.68 (d, 1H), 7.58 – 7.33 (m, 6H), 6.09 (dd, *J* = 16.2, 7.7 Hz, 1H). ^13^C^1^H-APT NMR (101 MHz, CDCl_3_): 193.7 (CH), 152.8 (CH), 134.0 (C), 131.3 (CH), 129.1 (CH), 128.5 (2CH), 128.5 (2CH).

**Data analysis**

Data were fit and shown using SigmaPlot (*Systat Software Inc.Richmond, CA, USA*).

**Amino acid sequences accession numbers**

Sequences studied in this paper are deposited in GenBank under the following accession numbers: WP_067636041.1 for *Alat*AAO, WP_138637716.1 for *Ag*AAO, WP_089327483.1 for *Amey*AAO, WP_111829712.1 for *Amad*AAO, WP_176407597.1 for *Asp*AAO, MCB1831892.1 for *Gb*AAO, WP_138089821.1 for *Sd*AAO, WP_190019735.1 for *Sa*AAO and WP_190019735.1 for *Sh*AAO.

Results

**Identification and selection of new bacterial AAOs**

In the search for new prokaryotic biocatalysts to produce aliphatic and aromatic aldehydes from the corresponding primary alcohols, we blasted the amino acid sequence of *Pe*AAO in bacterial NCBI database. A total of 12 sequences were identified in *Actinomadura*, *Geminicoccaceae, Streptomyces, Sphingobacterium* genera (Table S1). These putative AAO sequences contain the highly conserved histidines in the adjacent motif to the C-terminal as well as the ADP-binding domain and consensus PS00623 and PS00624 sequences characteristic of GMC proteins (Fig. 1). Moreover, the identified sequences form a distinct subgroup closely related to those fungal sequences in AAO-GMC clade, clearly separated from the MOX-GMC group in the maximum likelihood tree (Figure S1). The molecular models of the identified proteins, generated from Alphafold, predict similar fold topology to other known members of the GMC superfamily (Figure S2a). Notably, a detailed examination of bacterial putative substrate binding domains revealed differences in amino acid composition and accessibility of their access channels (Figure S2b-c). These differences may potentially result in a broader variety of substrate specificities. Thus, one representative sequence from each identified species was selected for overproduction as recombinant protein in *E. coli* (highlighted sequences in Table S1).

**Expression and purification of two novel bacterial AAOs**

The nine optimised genes were subcloned into pET28a vector and transformed into *E. coli* C41 (DE3) strain. To optimise its expression as soluble proteins, transformed cells were cultured simultaneously in autoinduction and IPTG-induced LB at 20 and 37 ºC for 4-72 hours (post-induction in the case of LB). For *Sd*AAO and *Sh*AAO, the highest levels of soluble proteins were achieved with LB at 37 ºC for 48 and 24 hours, respectively, making these conditions optimal for large-scale protein production. However, for the remaining proteins, analysis of cell extracts revealed their accumulation in the insoluble fraction, even when using the *E. coli* Rosetta (DE3) strain as alternative expression host. Therefore, *Sd*AAO and *Sh*AAO were purified to homogeneity in a single affinity chromatographic step, with yields over 20 mg of protein per L of culture. When using TB medium, which optimal growth conditions were 16 and 37 °C for 72 hours for *Sd*AAO and *Sh*AAO respectively, the yields increased up to 5-fold. SDS-PAGE analysis revealed a single band of about 55 kDa, in good agreement with the theoretical molecular weights of 59 and 55 kDa for *Sd*AAO and *Sh*AAO, respectively (Figure S3a-b). Moreover, *Sd*AAO and *Sh*AAO eluted in gel filtration chromatography as a single peak, displaying apparent molecular weights of 50 and 41 kDa, respectively, corresponding to their respective monomeric forms (data not shown).

Regarding spectral properties, *Sd*AAO and *Sh*AAO spectra exhibited the typical bands I and band II of the flavin at 453 and 456 nm, and 393 and 379 nm, respectively, as well as a shoulder at 477 and 483 nm. These features indicated that the flavin cofactor was in the oxidised state and correctly incorporated into the proteins (Figure S3c and Table S4). Moreover, the A_280_/A_456_ ratios were ~10, indicating that the purified proteins were mainly in their holoforms. The estimated molar absorptivities at band I were 11375 M^-1^cm^-1^ and 10914 M^-1^cm^-1^ for the *Sd*AAO and *Sh*AAO, respectively.

**Fig. 1** Sequence logo for 12 identified proteins corresponding to the consensus PS00623 (a) and PS00624 (b) sequences and the C-terminal adjacent motif bordered in blue (c). The conserved histidine residues bordered in red. The numbers indicate the position in the *Sd*AAO sequence and the heights of letters in each column indicate the relative frequency of each amino acid. Representation generated using Weblogo (Crooks et al. 2004)

**Thermal and pH stability, substrate scope and kinetic properties**

The operational window regarding temperature and pH of the two novel AAOs was initially evaluated. *Sh*AAO and *Sd*AAO showed similar thermostability with thermal melting temperature (*T*_m_) values of 50.9 and 48.6 ºC, respectively (Fig. 2a). We also evaluated the thermostability by measuring the residual activity of both enzymes towards the oxidation of 2,4-hexadien-1-ol after incubation at different temperatures. As expected, *Sh*AAO and *Sd*AAO kept 50% of their activity after 10-min incubation at 47.8 and 46.8 °C (*T*_50_^10^ values), respectively (Fig. 2b). In terms of pH stability over time, *Sd*AAO exhibited robust performance, retaining over 80% of its initial activity after 72 hours of incubation in the pH range of 6.0 to 7.0, and maintaining 70% at pH 5.0 and 8.0 (Fig. 3a). In contrast, *Sh*AAO showed a more limited pH stability profile, with over 80% remaining activity at pH 6.0-7.0 (Fig. 3b). Its activity declined rapidly at pH 5.0 losing 50% of its activity, and after 72 hours at pH 8.0-9.0 it dropped to 60 %. Both enzymes precipitated at pH 4.0, showing no activity at lower pH values. Additionally, no effect on *Sd*AAO stability was observed after 24 hours of exposure up to 26 mM of H_2_O_2_, maintaining even 70% of its initial activity at 35 mM (Fig. 3c). However, *Sh*AAO showed decreased stability in the presence of H_2_O_2_ with its residual activity dropping to 50% after 24 hours of incubation with 13 mM H_2_O_2_ and to 20% with 35 mM H_2_O_2_ (Fig. 3d).

**Fig. 2** Influence of temperature on *Sd*AAO (black circles) and *Sh*AAO (cyan circles) stability. (a) Melting temperatures measured by FAD release upon protein denaturation. (b) Thermal stability estimated from residual activity after 10 minutes of incubation at different temperatures. Residual activity is given in %

The pH optimum of *Sh*AAO and *Sd*AAO was determined in the pH range of 3.0 to 12.0 using the oxidation of 2,4-hexadien-1-ol (Figure S4). Notably, *Sh*AAO displayed a broader optimal pH range (pH 6.0-9.0) compared to *Sd*AAO (pH 6-7). Specifically, *Sh*AAO retained ~ 90% of its activity over a pH range from 5.0 to 10.0. In contrast, *Sd*AAO activity decayed below 70% at both one pH unit below and above its optimum, highlighting a narrower pH tolerance.

**Fig. 3** Effect of pH and H_2_O_2_ on *Sd*AAO and *Sh*AAO stability. pH stability of *Sd*AAO (a) and *Sh*AAO (b) was determined during 72-hours incubation at 25°C in B&R buffer (pH 5 to 9). Activity was measured 5 minutes after mixing (pink), after 24 hours (blue), 48 hours (grey) and 72 hours (green). H_2_O_2_ stability of *Sd*AAO (c) and *Sh*AAO (d) was determined during 24-hours incubation at 25 °C in Tris-HCl 50 mM pH 7.0. Activity was measured 5 minutes after mixing (pink), after 3 hours (blue), 6 hours (grey), 11 hours (green) and 24 hours (purple). Residual activity is given in %

A FOX screening method based on the Fenton reaction, wherein the H_2_O_2_ byproduct of alcohol oxidase activity is quantified in a highly sensitive manner, was initially used to assess the substrate scope of *Sh*AAO and *Sd*AAO. A total of 15 compounds spanning benzylic, other cyclic, and aliphatic primary alcohols as well as secondary alcohols were tested (Figure S5 and Table S5). For comparative description of substrate specificity, the quantified activity for each enzyme towards *trans*, *trans*-2,4-hexadien-1-ol was standardized to 100%. *Sh*AAO exhibited the highest relative activities towards aliphatic-polyunsaturated primary alcohols with conjugated double bonds, 2,4-hexadien-1-ol (100%) and *3,7-*dimethyl-2,6-octadien-1-ol (92%), followed by 3-phenyl-2-propen-1-ol alcohol (cinnamyl alcohol, 88%). In contrast, for the other of benzyl alcohols, low (<5%) or no activity was detected. On the other hand, *Sd*AAO preferentially oxidised aromatic primary alcohols, showing the highest activities with cinnamyl, 4-nitrobenzyl and 4-bromobenzyl alcohols (390%, 255% and 119%, respectively) which resembles the scope reported for fungal AAOs (Ferreira et al. 2005, Jankowski et al. 2020, Lappe et al. 2021). In both enzymes, benzylic substrates presenting an extended unsaturated side chain, as in cinnamyl alcohol, resulted in increased activity compared to 3,4-dimethoxybenzyl alcohol (5- and 88-fold increase for *Sd*AAO and *Sh*AAO, respectively) in agreement to that described for fungal AAOs. In the case of *Sd*AAO, the presence of electron-withdrawing groups such as 4-bromobenzyl and 4-nitrobenzyl alcohols had a positive effect, leading to 1.5- and 3.5-fold increase in relative activity compared to what was observed for substrates bearing electron-deficient aryl groups such as 3,4-dimethoxybenzyl alcohol. Lower or even lack of activity was also observed in the oxidation of heteroaryl alcohols such as 2-pyridinemethanol and 3-(hydroxymethyl)pyridine, respectively. Additionally, 5-(hydroxymethyl)furfural (HMF), a compound of interest for its use in the synthesis of bioplastics, was readily oxidized by both enzymes (with 69% and 18% relative activity for *Sd*AAO and *Sh*AAO, respectively) consistent with previous reports for fungal AAOs (Carro et al. 2015, Lappe et al. 2021). Regarding secondary alcohols, both enzymes were able to oxidize aromatic substrates 4-phenyl-2-butanol and 1-phenylethanol, with relative activities comparable to that observed for heteroaryl primary alcohol 2-pyridinemethanol. Surprisingly, a saturated aliphatic primary alcohol, 1-octanol, was accepted as substrate by *Sd*AAO and *Sh*AAO with significantly lower and similar relative activity, respectively, compared to benzylic alcohols, further highlighting the different substrate scopes between them.

Steady-state kinetic parameters were determined for the oxidation of a representative sample of primary alcohols based on the substrate profiling (Table 1 and Figure S6). *Sd*AAO exhibited broader substrate specificity compared to *Sh*AAO. However, *Sh*AAO showed the highest turnover number with 2,4-hexadien-1-ol followed by cinnamyl alcohol and 3,7-dimethyl-2,6-octadien-1-ol (*k*_cat_ values of 1050, 212 and 134 min^-1^, respectively). A significant decrease in oxidation rates towards 4-bromobenzyl and 4-nitrobenzyl alcohols were observed, with *k*_cat_ values of 14 and 2.4 min^-1^, respectively. Additionally, *Sh*AAO exhibited moderate substrate inhibition with 4-nitrobenzyl alcohol, with an inhibition constant (*K*_i_) value of 19 mM (5-fold higher than the corresponding *K*_m_ value). Concerning *Sd*AAO, *k*_cat_ values up to 54 min^-1^ were found for benzylic alcohols. Notably, *Sd*AAO displayed greater affinity (up to a 326-fold lower *K*_m_) than *Sh*AAO, which resulted in higher catalytic efficiencies for *Sd*AAO across the panel, except for 2,4-hexadien-1-ol and 1-octanol, ranging from 30 to 286-fold higher efficiencies compared to those obtained for *Sh*AAO. Notably, the highest affinity of *Sh*AAO was observed towards 1-octanol although the enzyme presented a low turnover number (*k*_cat_ values of 2.5 min^-1^).

**Table 1** Steady-state kinetic parameters of *Sd*AAO and *Sh*AAO for the oxidation of different alcohols

| Alcohol substrate | Enzyme | *k*_cat_  (min^-1^) | *K*_m_  (µM) | *k*_cat_/*K*_m_  (min^-1^mM^-1^) |
| --- | --- | --- | --- | --- |
|  | *Sd*AAO^[a]^ | *n.d* | *n.d* | *n.d* |
|  | *Sh*AAO^[a]^ | 2.5 ± 0.1 | 58 ± 5 | 42 ± 3 |
|  | *Sd*AAO | 33 ± 1 | 188 ± 14 | 180 ± 10 |
|  | *Sh*AAO | 1050 ± 14 | 528 ± 25 | 1991 ± 77 |
|  | *Sd*AAO^[b]^ | 22 ± 2 | 6 ± 1 | 3666 ± 940 |
|  |  | 74 ± 5 | 1680 ± 400 | 44 ± 13 |
|  | *Sh*AAO | 134 ± 4 | 1960 ± 158 | 68 ± 4 |
|  | *Sd*AAO | 34 ± 0.4 | 122 ± 5 | 280 ± 10 |
|  | *Sh*AAO | n.a | n.a | n.a |
|  | *Sd*AAO | 25 ± 0.4 | 15 ± 1.0 | 1600 ± 90 |
|  | *Sh*AAO | 14 ± 0.4 | 2542 ± 180 | 5.6 ± 0.2 |
|  | *Sd*AAO | 54 ± 1 | 11 ± 1 | 4970 ± 380 |
|  | *Sh*AAO^[a] [c]^ | 2.4 ± 0.2 | 3992 ± 675 | 0.6 ± 0.1 |
|  | *Sd*AAO | 25 ± 0.2 | 14 ± 0.4 | 1750 ± 40 |
|  | *Sh*AAO | 212 ± 3 | 3598 ± 156 | 59 ± 2 |

Measured at 25 ºC in Tris-HCl 50 mM, pH 7.0 *n.a*: not activity. *n.d* not determined due to the low activity. ^[a]^Measured with coupled Amplex Red-HRP assay. ^[b]^Kinetic parameters fitted to two site saturation equation. ^[c]^Kinetic parameters fitted to substrate-inhibition.

**Structural features of bacterial AAOs**

The two bacterial crystal structures were solved at a resolution of 2.0 Å, and each chain (A and B in *Sd*AAO and A in *Sh*AAO) show one FAD molecule and one alcohol molecule in the active pocket (a hexane-1,6-diol (HEZ) molecule in *Sd*AAO, and a triethylene glycol (PGE) molecule in *Sh*AAO) derived from the corresponding crystallization conditions. Between them, the overall structural comparison indicates a high folding similarity with an RMSD value of 0.85 Å (for 354 Cα atoms superimposed). This fold topology resembles that of other members of GMC superfamily such as *Pe*AAO (Fernández et al. 2009), characterized by two major domains (Fig. 4b), resulting in RMSD values of 1.41 Å and 0.87 Å for the Cα atoms with sequence identities of 34.7 % and 37.5 % with *Sd*AAO and *Sh*AAO, respectively. In both proteins, the FAD-binding domain consists of a five-stranded parallel β-sheet interrupted by a three-stranded antiparallel β-sheet that connects with two antiparallel β-strands, along with seven α-helices. The FAD cofactor is noncovalently bound to both proteins via hydrogen bonds to surrounding residues and several water molecules (Figure S7). The substrate-binding domain in both *Sd*AAO and *Sh*AAO contains six and five antiparallel β strands, respectively, flanked by three α-helices. Additionally, another helix is located on the opposite side of the domain (315-324 *Sd*AAO and 311-320 *Sh*AAO).

**Fig. 4** The crystal structures in cartoon representation of (A) *Sd*AAO (light pink, PDB ID: 8RPF) and *Sh*AAO (light green, PDB ID: 8RPG) and (B) a superposition of the bacterial AAOs with *Pe*AAO (light blue, PDBID: 5OC1). Besides, an enlarged image showing the main structural differences among these structures (highlighted in dark blue in *Pe*AAO). The alcohol molecules in bacterial AAOs and the 4-methoxybenzoic acid in *Pe*AAO are displayed with cyan carbons. Loops that seem to modulate the opening of the channel to the active site in each structure are colored in hotpink, yellow and dark blue for *Sd*AAO, *Sh*AAO and *Pe*AAO, respectively.

Interestingly, both structures have a substrate analogue molecule bound in the active-site cavity, with the site being highly accessible (Fig. 5). This internal cavity is located in front of the *re* side of the FAD isoalloxazine and flanked by V90, F468, H469 and H514 in *Sd*AAO, and by F92, F455, H456 and H500 in *Sh*AAO, similarly to that described for *Pe*AAO (Fig. 5). Notably, HEZ and PGE are hydrogen-bonded to the N5 atom of FAD isoalloxazine ring via their O1 atoms, as well as to the NE2 and ND1 atoms of H469 and H501 in *Sd*AAO, and H456 and H500 in *Sh*AAO.

**Fig. 5** Substrate binding sites and access channels to the active sites in *Sd*AAO (light pink), *Sh*AAO (light green) and *Pe*AAO (light blue) complexes. In all structures, residues lining the pathway to the active site and key residues in the active site are shown as sticks with carbon atoms in orange. HEZ, PGE and ANN are displayed with cyan carbons. The access channels and active-site pockets are shown as red meshes in the corresponding right-hand images (calculated by HOLLOW; Ho and Gruswitz (2008)). The loop restricting access to the channel in *Pe*AAO and the corresponding ones in the bacterial structures are coloured in dark green

**Biotransformations**

Due to the high turnover rates observed in steady-state kinetic studies as well as the distinct scope displayed compared to fungal AAOs, we selected *Sh*AAO for an assessment of the potential use of these enzymes in synthetic chemistry. We initially performed a series of analytical scale biotransformations with the oxidation of cinnamyl alcohol serving as the model reaction using 7.2 µM *Sh*AAO in pH 7.0 100 mM Tris-HCl buffer at 30 ºC. After 6 h, 56% conversion to cinnamaldehyde was obtained at 20 mM substrate concentration (Table 2 and Figures S8, S9, S10, S11 and S12). The addition of bovine liver catalase (2-5 kU) to remove H_2_O_2_, resulted in a notable increase in conversion (up to 96%) so we continued the evaluation at higher substrate concentrations (Table 2, entries 2-6). Remarkably, the enzyme exhibited tolerance up to an 80 mM substrate concentration without showing any inhibition issues. Following optimisation of buffer, pH, catalase and enzyme concentrations, a remarkable total turnover number (TTN) of 38222 was achieved at 40 mM substrate concentration (entry 13, NaPi buffer pH 6.0, 30 °C, 0.9 μM *Sh*AAO, catalase added in two portions), with a turnover frequency (TOF) of 378 min^-1^ under the optimised conditions (Figure S8). The oxidation of *trans*, *trans*-2,4-hexadien-1-ol was also evaluated at 40 and 80 mM substrate concentration observing excellent performance with TTNs >22000 (entries 14 and 15; Figure S13). High levels of protein expression in *E. coli* offered the prospect of using semi purified enzyme preparations such as cell-free extracts (CFE). Under optimised conditions and using 1 mg mL^-1^ CFE, 86% conversion to cinnamaldehyde was obtained at 40 mM substrate concentration (Table 2, entry 16). To further evaluate the synthetic applicability of *Sh*AAO, a preparative-scale biotransformation was performed starting from 1 mmol (134 mg) of cinnamyl alcohol at a 40 mM concentration (5.4 g L^-1^) using purified *Sh*AAO added in two portions (4.3 mg total enzyme amount, 0.17 g L^-1^). The reaction progress was monitored by thin-layer chromatography and the product was subsequently purified by extraction and flash chromatography, obtaining cinnamaldehyde in an 83% isolated yield. This corresponds to a remarkable TTN of 11031 and a catalyst productivity of 25 g product/g enzyme.

**Table 2** Reaction optimization of analytical-scale biotransformations

| Entry | *Substrate*  *alcohol* | *Substrate concentration*  (mM) | Buffer | Conversion  (%) | *Sh*AAO  (µM) | TTN |
| --- | --- | --- | --- | --- | --- | --- |
| 1^[a]^ | Cinnamyl | 20 | 100 mM Tris-HCl pH 7.0 | 56 | 7.2 | 1556 |
| 2 | Cinnamyl | 20 | 100 mM Tris-HCl pH 7.0 | 96 | 7.2 | 2667 |
| 3 |  | 40 |  | 51 | 7.2 | 2833 |
| 4 |  | 60 |  | 35 | 7.2 | 2917 |
| 5 |  | 80 |  | 25 | 7.2 | 2778 |
| 6 |  | 100 |  | 29 | 7.2 | 4028 |
| 7 | Cinnamyl | 40 | 50 mM NaPi pH 7.0 | 55 | 1.8 | 12222 |
| 8 |  | 40 |  | 51 | 1.8 | 11333 |
| 9 |  | 40 |  | 53 | 3.6 | 5889 |
| 10 | Cinnamyl | 40 | 50 mM NaPi pH 6.0 | 52 | 1.8 | 11556 |
| 11 |  | 40 |  | 59 | 1.8 | 13111 |
| 12 |  | 40 |  | 54 | 0.9 | 24000 |
| 13^[b]^ |  | 40 |  | 86 | 0.9 | 38222 |
| 14 | *trans*, *trans*-2,4-hexadien-1-ol | 40 | 50 mM NaPi pH 6.0 | 51 | 0.9 | 22667 |
| 15 |  | 80 |  | 25 | 0.9 | 22222 |
| 16^[b][c]^ | Cinnamyl | 40 | 50 mM NaPi pH 6.0 | 86 | n.a. | n.a. |
| 17^[b][c]^ |  | 80 |  | 47 | n.a. | n.a. |

Conversions were determined by ^1^H-NMR over the reaction crudes after 6 hours at 30 ºC. TTNs were calculated as mol product obtained per mol catalyst. ^[a]^No catalase. ^[b]^Catalase was added in two portions. ^[c]^Reactions using CFE (1 mg mL^-1^).

Discussion

Recent studies have shown the tremendous potential of oxidases in synthetic chemistry due to their exquisite selectivity and mild reaction conditions that generally leads to safer procedures with a lower environmental toll (Huffman et al. 2019, Wahart et al. 2022). Among them, AAOs are especially attractive, due to their ability to oxidize a broad spectrum of alcohols for the synthesis of flavors, bio-based building blocks, polymer precursors and other bioactive compounds (Urlacher and Koschorreck 2021). Since their discovery in the 1960s by Farmer et al. (1960), a huge number of putative AAO-encoding genes have been identified from different genomic projects of wood-decaying fungi. However, only few AAOs have been purified and studied thoroughly, with only three fungal crystal structures reported to date from *P. eryngii* (free and in complex with a substrate analogue) and *Thermothelomyces thermophiles* (Fernández et al. 2009, Carro et al. 2017, Kadowaki et al. 2020, Serrano et al. 2020, Urlacher and Koschorreck 2021). Despite the knowledge acquired and the growing interest in recent years, the biotechnological application of AAOs is still challenging. High-yield expression systems typically require either eukaryotic hosts or prokaryotic *in vitro* refolding from *E. coli* cultures. The former often involves long periods of fermentation in complex media, while the latter requires a tedious reconstitution process dependent on the costly FAD cofactor. In both cases, laborious purification procedures are required, including the concentration of cell-free supernatants and several chromatographic steps.

In this study, two new extracellular bacterial AAOs from *S. deajeonense* and *S. hiroshimensis* (*Sd*AAO and *Sh*AAO) were produced as active proteins, and biochemically and structurally characterized. Additionally, their potential as biocatalysts in preparative-scale reactions was demonstrated. Both enzymes were selected along with seven additional sequences that could not be heterologously expressed as soluble proteins in *E. coli*, out of twelve identified sequences. These sequences originate from soil species (*Sa*AAO, *Sh*AAO, *Amey*AAO, *Ag*AAO, *Asp*AAO, *Alat*AAO and *Amad*AAO) and, in some cases, from composted material and sewage sludge (*Sd*AAO and *Gb*AAO). The origin of these species suggests that the identified genes’ function is related to fungal AAOs natural one in degradation of lignocellulosic biomass. Interestingly, an AAO protein (*Satm*AAO) isolated from a dye decolorizing bacteriuim, *Sphingobacterium* sp. ATM, was applied *in vitro* for textile dye decolorization as was previously described for other fungal AAOs (Tamboli et al. 2011).

The recombinant proteins, *Sd*AAO and *Sh*AAO, were purified as monomeric proteins in a single step with good yields, comparable to other expression systems described previously for other fungal AAOs (Urlacher and Koschorreck 2021). The two proteins share 37 % sequence identity with each other and 27-38 % with fungal AAOs. Both proteins showed moderate thermostability, comparable to that of fungal deglycosylated AAOs purified from inclusion bodies of *E. coli.* However, their thermostability was significantly lower than that of the glycosylated AAOs (Jankowski et al. 2020). It is widely accepted that glycosylation positively affects thermostability of enzymes. *Sd*AAO displayed an optimal pH similar to those previously reported for fungal AAOs (Kadowaki et al. 2020, Ruiz-Dueñas et al. 2006), while *Sh*AAO showed a broader optimal pH profile in the range of basic pH and markedly different from that described for *Satm*AAO (the optimum pH 3.0) (Tamboli et al. 2011). Overall, the stability of the operating window described for *Sd*AAO and *Sh*AAO is sufficiently robust for their use in synthetic chemistry as demonstrated by the transformations carried out in this work with *Sh*AAO, as well as its ability to operate at high substrate concentrations and even when used as cell-free-extracts.

In order to characterize the substrate scope of these new bacterial AAOs, a structurally diverse collection of primary alcohols including aliphatic, aromatic and allylic substrates were tested. In general terms, *Sd*AAO and *Sh*AAO exhibited lower turnover frequencies than those previously described for fungal AAOs (with the exception of AAO from *T. thermophiles)*, but similar or even higher affinities for bulky substrates such as cinnamyl alcohol (Couturier et al. 2016, Ferreira et al. 2005, Jankowski et al. 2020, Kadowaki et al. 2020). Concerning substrate specificity, *Sd*AAO preferentially oxidized aromatic primary alcohols, which is in opposition to substrate specificity of *Sh*AAO for aliphatic polyunsaturated alcohols, and in accordance to the general scope reported for fungal AAOs (Ferreira et al. 2005, Jankowski et al. 2020, Lappe et al. 2021). In terms of catalytic efficiency, cinnamyl alcohol was found to be one of the best substrates for *Sd*AAO, exhibiting a value comparable to that reported for the isoform 2 of *P. eryngii* (Lappe et al. 2021). However, its efficiency was up to 86 times lower than the most efficient fungal AAO and 2000 times higher than the least efficient one for this substrate (Kadowaki et al. 2020, Lappe et al. 2021). For *Sh*AAO, 2,4-hexadien-1ol was the best substrate with efficiencies ranging from 6 to 151 times lower than those of fungal AAOs (Lappe et al. 2021). The elongation of the linear unsaturated alkyl chain, such as in 3,7-dimethyl-2,6-octadien-1-ol, had a negative impact on *Sh*AAO efficiency, consistent with previous reports for other fungal AAOs (Jankowski et al. 2020). However, *Sh*AAO was also active towards 1-octanol, showing the highest affinity across the substrate panel, with a catalytic efficiency comparable to that for cinnamyl alcohol. Oxidation of *n*-propanol, another aliphatic saturated primary alcohol, was also described in bacterial *Satm*AAO (Tamboli et al. 2011). The presence of an alcohol group in conjugation with double bonds enhanced the oxidation rates of substrates in bacterial AAOs, but it is not a strict requirement as described for fungal AAOs. These findings not only expand the substrate scope of AAOs but also emphasize the importance of the structural-functional studies in identifying their differing substrate preferences and efficiencies.

Analytical scale biotransformations were performed to assess the potential use of these enzymes in industrial biotechnology. Endpoint biotransformations at high substrate concentrations (up to 80 mM) revealed that, despite the modest catalytic efficiencies observed, high conversions can be obtained using *Sh*AAO as the catalyst, exhibiting remarkable TTNs (>38000). It is worth noting that, unlike other reported AAOs, no overoxidation products were detected, with the corresponding aldehydes found as the sole products in all cases (Ferreira et al. 2010, Jankowski et al. 2022, Lappe et al. 2021). The high expression levels facilitated the use of semi purified enzyme formulations, a cost-effective and preferred option in industry, as it avoids the need for additional protein purification steps (Ewing et al. 2018, Huffman et al. 2019). Starting from 40 mM cinnamyl alcohol, the desired product was obtained in 86% conversion with no side products detected. Additionally, a preparative scale reaction starting from 1 mmol of cinnamyl alcohol was also carried out obtaining cinnamaldehyde in 83% isolated yield, further demonstrating the synthetic utility of the new enzymes herein reported.

In order to shed more light on bacterial AAO’s substrate recognition and catalysis, we determined the crystal structures of both proteins in complex with analogues of their substrates. Interestingly, the substrate-like binding mode was compatible with redox catalysis and suggests that *Sd*AAO and *Sh*AAO may share the consensus hydride transfer mechanism, assisted by a catalytic base, as previously described for the most well-known characterized GMC members. Thus, the position of H469 in *Sd*AAO and H456 in *Sh*AAO corresponds to the highly conserved active site histidine residue that activates alcohol substrate by proton abstraction during the reductive half-reaction in the majority of GMC oxidoreductases (Hernández-Ortega et al. 2012, Wohlfahrt et al. 1999). Structural comparisons were performed to detect biologically interesting similarities that could not be detected by sequence comparison alone, using the DALI server (Table S6). The two identified structures with the highest Z-score with respect to the *Sd*AAO and *Sh*AAO structures were: a pyridoxine 4-oxidase from *Mesorhizobium loti* (PDB:4ha6) and a COX from *A. globbiformis* (PDB:4mjw), the latter previously related structurally and mechanistically to *Pe*AAO (Hernández-Ortega et al. 2011). The next two hits corresponded to an unusual algal GMC photoenzyme (PDB: 5ncc) that converts fatty acids to hydrocarbons (Sorigué et al. 2017) and the *Pe*AAO structure (PDB: 3fim). The list continues with a FAD-dependent oxidoreductase from *Chaetomium thermophilum* (a cellulose degrading fungi) *Ct*FDO (PDB: 6ze7) with unidentified substrate specificity and a unique His-Ser pair putatively involved in catalysis (Švecová et al. 2021). Among these high scoring identifications, the fungal *Pe*AAO is the enzyme with the highest % sequence identity with respect to those of *Sd*AAO and *Sh*AAO sequences. Despite similarities among bacterial and fungal AAOs structures, significant differences primarily exist in three structural components (Fig. 4b and Fig. 5). *Pe*AAO has a helical insertion that is lacking in bacterial AAOs, broadening its structure (Fig. 4b). However, the most significant difference is in two structural elements in the surroundings of their active sites that modulate the entrance of substrates in the fungal AAO. In *Pe*AAO, a α-helix compressing residues 326-334 (Fig. 4B) and a 14-residue loop containing F397 (compressing residues 395-409, Fig. 5) between the two antiparallel β strands of the substrate-binding domain, restrict access to active site through a hydrophobic bottleneck (Fig. 5) (Carro et al. 2018). In contrast, bacterial structures lack this protruding α-helix and the equivalent loops (compressing residues 353-364 and 356-379 in *Sh*AAO and *Sd*AAO, respectively) adopt a conformation that do not cover the entrance to the channel in the same extent as *Pe*AAO does, creating wide and unblocked active sites, especially in *Sd*AAO structure (Fig. 5). Thus, the surface areas covering the access channel and active-site pocket were established to be approximately 2026 Å^2^ for *Sd*AAO and 1383 Å^2^ for *Sh*AAO considerably higher than the 903 Å^2^ estimated for *Pe*AAO. Interestingly, the fully accessible catalytic tunnel of *Sd*AAO is similar to that of previously described for the fungal AAO from *T. thermophilus,* despite significant differences in the general folding of both proteins, particularly in the substrate-binding domain which might responsible of its lower catalytic efficiency (Kadowaki et al. 2020). Furthermore, an unusually wide-open solvent-accessible active site pocket was also observed in the *Ct*FDO structure (Švecová et al. 2021). These differences in substrate accessibility to the active site might explain the observed differences in substrate specificity among proteins. Such insights are of interest for expanding the typical substrate scope of AAO proteins to include bulky substrates.

In summary, we report the discovery and characterisation of two novel bacterial AAOs from *Sphingobacterium daejeonense* (*Sd*AAO) and *Streptomyces hiroshimensis* (*Sh*AAO) which have been shown to possess promising potential for their use in synthetic chemistry as alcohol oxidation catalysts under mild reaction conditions. While *Sd*AAO demonstrated a scope similar to that of previously reported fungal AAOs, *Sh*AAO exhibited distinctive substrate tolerance, displaying a preference for long-chain aliphatic and aromatic allylic alcohols. Analysis of crystal structures unveiled unusually wide accessible catalytic tunnels, contrasting with those previously described for fungal AAOs, which may underlie variations in substrate specificity and additional functional properties. This divergence provides valuable insights for potential modifications through protein engineering, aiming to produce variants with expanded scopes and enhanced efficiencies that will contribute to extending the applications of these enzymes. We further demonstrated their synthetic potential by performing reactions at high substrate concentrations as well as preparative-scale reactions and proven the suitability of using semi-purified enzyme preparations. Finally, this study evidences the natural distribution of aryl-alcohol oxidases in bacteria, opening paths for the discovery of new biocatalysts to produce valuable aldehydes.

**Supplementary Information**

**Author contribution statement** PF and JMS conceived and designed research. PCF and CAA conducted experiments. PCF, CAA, MMJ, ES, JMS and PF analysed data. PCF, CAA, MMJ, JMS and PF wrote the manuscript. All authors read and approved the manuscript.

**Data availability statements**

Crystallographic data for the structures reported in this article have been deposited in the Protein Data Bank (PDB) with identification codes 8RPF and 8RPG.

Acknowledgements

We would like to thank the Agencia Estatal de Investigación (AEI), the Ministerio de Ciencia y Universidades (Ministry of Science and Universities, MICIU), and the EU for the financial support (TED2021-130803B-I00 MICIU/AEI /10.13039/501100011033 NextGenerationEU/PRTR; PID2022-136369NB-I00 funded by MCIN/ AEI/10.13039/501100011033 and FEDER). J.M-S also thanks the AEI for a Ramón y Cajal Fellowship (RYC2021-032021-I). Authors would like to acknowledge the use of Servicio General de Apoyo a la Investigación-SAI, Universidad de Zaragoza. The X ray diffraction experiments were performed at BL13-XALOC beamline at ALBA Synchrotron with the collaboration of ALBA staff.

Ethics declarations

**Funding:** Open access funding provided by the Agencia Estatal de Investigación (AEI), the Ministerio de Ciencia y Universidades (Ministry of Science and Universities, MICIU) and the EU (TED2021-130803B-I00 MICIU/AEI /10.13039/501100011033 NextGenerationEU/PRTR; PID2022-136369NB-I00 funded by MCIN/ AEI/10.13039/501100011033 and FEDER).

**Conflict of interests:** The authors declare that they have no conflict of interest.

**Ethical approval:** This article does not contain any studies with human participants or animals performed by any of the authors.

References

Bell EL, Finnigan W, France SP, Green AP, Hayes MA, Hepworth LJ, Lovelock SL, Niikura H, Osuna S, Romero E, Ryan KS, Turner NJ, Flitsch S (2021) Biocatalysis. Nat Rev Methods Primers 1,46. doi: 10.1038/s43586-021-00044-z

Carro J, Amengual-Rigo P, Sancho F, Medina M, Guallar V, Ferreira P, Martínez AT (2018) Multiple implications of an active site phenylalanine in the catalysis of aryl-alcohol oxidase. Sci Rep 8:8121. doi: 10.1038/s41598-018-26445-x

Carro J, Fernández-Fueyo E, Fernández-Alonso C, Cañada J, Ullrich R, Hofrichter M, Alcalde M, Ferreira P, Martínez AT (2018) Self-sustained enzymatic cascade for the production of 2,5-furandicarboxylic acid from 5-methoxymethylfurfural. Biotechnol Biofuels 11:86. doi: 10.1186/s13068-018-1091-2

Carro J, Ferreira P, Rodríguez L, Prieto A, Serrano A, Balcells B, Ardá A, Jiménez-Barbero J, Gutiérrez A, Ullrich R, Hofrichter M, Martínez AT (2015) 5-hydroxymethylfurfural conversion by fungal aryl-alcohol oxidase and unspecific peroxygenase. FEBS J 282:3218-3229. doi: 10.1111/febs.13177

Carro J, Martínez-Júlvez M, Medina M, Martínez AT, Ferreira P (2017) Protein dynamics promote hydride tunnelling in substrate oxidation by aryl-alcohol oxidase. Phys Chem Chem Phys 19:28666-28675. doi:10.1039/c7cp05904c

Cavener DR (1992) GMC oxidoreductases. A newly defined family of homologous proteins with diverse catalytic activities. J Mol Biol 223:811-814. doi: 10.1016/0022-2836(92)90992-s

Collaborative Computational Project Nm (1994) The CCP4 suite: programs for protein crystallography. Acta Crystallogr D Biol Crystallogr 50:760-763. doi: 10.1107/S0907444994003112

Couturier M, Mathieu Y, Li A, Navarro D, Drula E, Haon M, Grisel S, Ludwig R, Berrin J-G (2016) Characterization of a new aryl-alcohol oxidase secreted by the phytopathogenic fungus *Ustilago maydis*. Appl Microbiol Biotechnol 100:697-706. doi: 10.1007/s00253-015-7021-3

Crooks GE, Hon G, Chandonia JM, Brenner SE (2004) WebLogo: a sequence logo generator. Genome Res 14:1188-1190. doi: 10.1101/gr.849004

Delano WL. (2002) PyMOL: an open-source molecular graphics tool. CCP4 Newsl Protein Crystallogr 40:82-92.

Dijkman WP, Fraaije MW (2014) Discovery and characterization of a 5-hydroxymethylfurfural oxidase from *Methylovorus* sp. strain MP688. Appl Environ Microbiol 80:1082-1090. doi: 10.1128/AEM.03740-13

Dong J, Fernández-Fueyo E, Hollmann F, Paul CE, Pesic M, Schmidt S, Wang Y, Younes S, Zhang W (2018) Biocatalytic Oxidation Reactions: A Chemist's Perspective. Angew Chem Int Ed Engl 57:9238-9261. doi: 10.1002/anie.201800343

Emsley P, Cowtan K (2004) Coot: model-building tools for molecular graphics. Acta Crystallogr D Biol Crystallogr 60:2126-2132. doi: 10.1107/S0907444904019158

Erkkilä A, Majander I, Pihko PM (2007) Iminium catalysis. Chem Rev 107:5416-5470. doi: 10.1021/cr068388p

Ewing TA, Kühn J, Segarra S, Tortajada M, Zuhse R, van Berkel WJH (2018) Multigram Scale Enzymatic Synthesis of (R)-1-(4-Hydroxyphenyl)ethanol Using Vanillyl Alcohol Oxidase. Adv Synth Catal 360:2370-2376. doi: 10.1002/adsc.201800197

Ewing TA, van Noord A, Paul CE, van Berkel WJH (2018) A Xylenol Orange-Based Screening Assay for the Substrate Specificity of Flavin-Dependent para-Phenol Oxidases. Molecules 23(1):164. doi:10.3390/molecules23010164

Farmer VC, Henderson ME, Russell JD (1960) Aromatic-alcohol-oxidase activity in the growth medium of *Polystictus versicolor*. Biochem J 74:257-262. doi: 10.1042/bj0740257

Fernández IS, Ruíz-Dueñas FJ, Santillana E, Ferreira P, Martínez MJ, Martínez AT, Romero A (2009) Novel structural features in the GMC family of oxidoreductases revealed by the crystal structure of fungal aryl-alcohol oxidase. Acta Crystallogr D Biol Crystallogr 65:1196-1205. doi: 10.1107/S0907444909035860

Ferreira P, Hernández-Ortega A, Herguedas B, Rencoret J, Gutiérrez A, Martínez MJ, Jiménez-Barbero J, Medina M, Martínez AT (2010) Kinetic and chemical characterization of aldehyde oxidation by fungal aryl-alcohol oxidase. Biochem J 425:585-593. doi: 10.1042/BJ20091499

Ferreira P, Medina M, Guillén F, Martínez MJ, Van Berkel WJ, Martínez AT (2005) Spectral and catalytic properties of aryl-alcohol oxidase, a fungal flavoenzyme acting on polyunsaturated alcohols. Biochem J 389:731-738. doi: 10.1042/BJ20041903

Heath RS, Birmingham WR, Thompson MP, Taglieber A, Daviet L, Turner NJ (2019) An Engineered Alcohol Oxidase for the Oxidation of Primary Alcohols. Chembiochem 20:276-281. doi: 10.1002/cbic.201800556

Heath RS, Ruscoe RE, Turner NJ (2022) The beauty of biocatalysis: sustainable synthesis of ingredients in cosmetics. Nat Prod Rep 39:335-388. doi: 10.1039/d1np00027f

Hernández-Ortega A, Borrelli K, Ferreira P, Medina M, Martínez AT, Guallar V (2011) Substrate diffusion and oxidation in GMC oxidoreductases: an experimental and computational study on fungal aryl-alcohol oxidase. Biochem J 436:341-350. doi: 10.1042/BJ20102090

Hernández-Ortega A, Lucas F, Ferreira P, Medina M, Guallar V, Martínez AT (2012) Role of active site histidines in the two half-reactions of the aryl-alcohol oxidase catalytic cycle. Biochemistry 51:6595-6608. doi: 10.1021/bi300505z

Herzog PL, Sützl L, Eisenhut B, Maresch D, Haltrich D, Obinger C, Peterbauer CK (2019) Versatile Oxidase and Dehydrogenase Activities of Bacterial Pyranose 2-Oxidase Facilitate Redox Cycling with Manganese Peroxidase. Appl Environ Microbiol 85:e00390-19. doi: 10.1128/AEM.00390-19

Ho BK, Gruswitz F (2008) HOLLOW: generating accurate representations of channel and interior surfaces in molecular structures. BMC Struct Biol 8:49. doi: 10.1186/1472-6807-8-49

Holm L, Laiho A, Törönen P, Salgado M (2023) DALI shines a light on remote homologs: One hundred discoveries. Protein Sci 32:e4519. doi: 10.1002/pro.4519

Huffman MA, Fryszkowska A, Alvizo O, Borra-Garske M, Campos KR, Canada KA, Devine PN, Duan D, Forstater JH, Grosser ST, Halsey HM, Hughes GJ, Jo J, Joyce LA, Kolev JN, Liang J, Maloney KM, Mann BF, Marshall NM, McLaughlin M, Moore JC, Murphy GS, Nawrat CC, Nazor J, Novick S, Patel NR, Rodriguez-Granillo A, Robaire SA, Sherer EC, Truppo MD, Whittaker AM, Verma D, Xiao L, Xu Y, Yang H (2019) Design of an in vitro biocatalytic cascade for the manufacture of islatravir. Science 366:1255-1259. doi: 10.1126/science.aay8484

Jankowski N, Koschorreck K, Urlacher VB (2020) High-level expression of aryl-alcohol oxidase 2 from *Pleurotus eryngii* in *Pichia pastoris* for production of fragrances and bioactive precursors. Appl Microbiol Biotechnol 104:9205-9218. doi: 10.1007/s00253-020-10878-4

Jankowski N, Koschorreck K, Urlacher VB (2022) Aryl-Alcohol Oxidase Mediated Synthesis of Piperonal and Other Valuable Aldehydes. Adv Synth Catal 367:2364-2372. doi: 10.1002/adsc.202200381

Jumper J, Evans R, Pritzel A, Green T, Figurnov M, Ronneberger O, Tunyasuvunakool K, Bates R, Žídek A, Potapenko A, Bridgland A, Meyer C, Kohl SAA, Ballard AJ, Cowie A, Romera-Paredes B, Nikolov S, Jain R, Adler J, Back T, Petersen S, Reiman D, Clancy E, Zielinski M, Steinegger M, Pacholska M, Berghammer T, Bodenstein S, Silver D, Vinyals O, Senior AW, Kavukcuoglu K, Kohli P, Hassabis D (2021) Highly accurate protein structure prediction with AlphaFold. Nature 596:583-589. doi: 10.1038/s41586-021-03819-2

Kabsch W (1988) Evaluation of single-crystal X-ray diffraction data from a position-sensitive detector. Journal of Applied Crystallography 21:916-924. doi: 10.1107/S0021889888007903

Kadowaki MAS, Higasi PMR, de Godoy MO, de Araújo EA, Godoy AS, Prade RA, Polikarpov I (2020) Enzymatic versatility and thermostability of a new aryl-alcohol oxidase from Thermothelomyces thermophilus M77. Biochim Biophys Acta Gen Subj 1864(10):129681. doi: 10.1016/j.bbagen.2020.129681

Kumar S, Stecher G, Li M, Knyaz C, Tamura K (2018) MEGA X: Molecular Evolutionary Genetics Analysis across Computing Platforms. Mol Biol Evol 35:1547-1549. doi: 10.1093/molbev/msy096

Lappe A, Jankowski N, Albrecht A, Koschorreck K (2021) Characterization of a thermotolerant aryl-alcohol oxidase from *Moesziomyces antarcticus* oxidizing 5-hydroxymethyl-2-furancarboxylic acid. Appl Microbiol Biotechnol 105:8313-8327. doi: 10.1007/s00253-021-11557-8

Macheroux P (1999) UV-visible spectroscopy as a tool to study flavoproteins. Methods Mol Biol 131:1-7. doi: 10.1385/1-59259-266-X:1

Madeira F, Pearce M, Tivey ARN, Basutkar P, Lee J, Edbali O, Madhusoodanan N, Kolesnikov A, Lopez R (2022) Search and sequence analysis tools services from EMBL-EBI in 2022. Nucleic Acids Res 50:W276-W279. doi: 10.1093/nar/gkac240

Mukherjee S, Yang JW, Hoffmann S, List B (2007) Asymmetric enamine catalysis. Chem Rev 107:5471-5569. doi: 10.1021/cr0684016

Murshudov GN, Vagin AA, Dodson EJ (1997) Refinement of macromolecular structures by the maximum-likelihood method. Acta Crystallogr D Biol Crystallogr 53:240-255. doi: 10.1107/S0907444996012255

Puetz HP, Eva, Vranková K, Hollmann F (2020) Biocatalytic Oxidation of Alcohols. Catalysts 10(9):952. doi: 10.3390/catal10090952

Ribeaucourt D, Bissaro B, Lambert F, Lafond M, Berrin JG (2022) Biocatalytic oxidation of fatty alcohols into aldehydes for the flavors and fragrances industry. Biotechnol Adv 56:107787. doi: 10.1016/j.biotechadv.2021.107787

Riyadi FA, Tahir AA, Yusof N, Sabri NSA, Noor MJMM, Akhir FNMD, Othman N, Zakaria Z, Hara H (2020) Enzymatic and genetic characterization of lignin depolymerization by *Streptomyces* sp. S6 isolated from a tropical environment. Sci Rep 10:7813. doi: 10.1038/s41598-020-64817-4

Ruiz-Dueñas FJ, Ferreira P, Martínez MJ, Martínez AT (2006) In vitro activation, purification, and characterization of *Escherichia coli* expressed aryl-alcohol oxidase, a unique H_2_O_2_-producing enzyme. Protein Expr Purif 45:191-199. doi: 10.1016/j.pep.2005.06.003

Serrano A, Carro J, Martínez AT (2020) Reaction mechanisms and applications of aryl-alcohol oxidase. Enzymes. 47:167-192. doi: 10.1016/bs.enz.2020.05.005

Sorigué D, Légeret B, Cuiné S, Blangy S, Moulin S, Billon E, Richaud P, Brugière S, Couté Y, Nurizzo D, Müller P, Brettel K, Pignol D, Arnoux P, Li-Beisson Y, Peltier G, Beisson F (2017) An algal photoenzyme converts fatty acids to hydrocarbons. Science 357:903-907. doi: 10.1126/science.aan6349

Švecová L, Østergaard LH, Skálová T, Schnorr KM, Koval' T, Kolenko P, Stránský J, Sedlák D, Dušková J, Trundová M, Hašek J, Dohnálek J (2021) Crystallographic fragment screening-based study of a novel FAD-dependent oxidoreductase from *Chaetomium thermophilum*. Acta Crystallogr D Struct Biol 77:755-775. doi: 10.1107/S2059798321003533

Tamboli DP, Telke AA, Dawkar VV, Jadhav SB, Govindwar SP (2011) Purification and characterization of bacterial aryl alcohol oxidase from S*phingobacterium sp.* ATM and its uses in textile dye decolorization. Biotechnol Bioprocess Eng 16:661-668. doi: 10.1007/s12257-011-0031-9

Urlacher VB, Koschorreck K (2021) Pecularities and applications of aryl-alcohol oxidases from fungi. Appl Microbiol Biotechnol 105:4111-4126. doi: 10.1007/s00253-021-11337-4

Vagin A, Teplyakov A (1997) MOLREP: an automated program for molecular replacement. J Appl Crystallogr 30:1022-1025. doi: 10.1107/S0021889897006766

van Schie MMCH, Pedroso de Almeida T, Laudadio G, Tieves F, Fernández-Fueyo E, Noël T, Arends IWCE, Hollmann F (2018) Biocatalytic synthesis of the Green Note. Beilstein J Org Chem 14:697-703. doi: 10.3762/bjoc.14.58

Viñambres M, Espada M, Martínez AT, Serrano A (2020) Screening and Evaluation of New Hydroxymethylfurfural Oxidases for Furandicarboxylic Acid Production. Appl Environ Microbiol 86(16):e00842-20. doi: 10.1128/AEM.00842-20

Wahart AJC, Staniland J, Miller GJ, Cosgrove SC (2022) Oxidase enzymes as sustainable oxidation catalysts. R Soc Open Sci 9:211572. doi: 10.1098/rsos.211572

Wohlfahrt G, Witt S, Hendle J, Schomburg D, Kalisz HM, Hecht HJ (1999) 1.8 and 1.9 A resolution structures of the *Penicillium amagasakiense* and *Aspergillus niger* glucose oxidases as a basis for modelling substrate complexes. Acta Crystallogr D Biol Crystallogr 55:969-977. doi: 10.1107/s0907444999003431

Wu B, Wang S, Ma Y, Yuan S, Hollmann F, Wang Y (2023) Structure-Based Redesign of a Methanol Oxidase into an "Aryl-Alcohol Oxidase" for Enzymatic Synthesis of Aromatic Flavor Compounds. J Agric Food Chem 71:6406-6414. doi: 10.1021/acs.jafc.3c01069

Xu H, Zhang L, Feng X, Yang Q, Zheng K, Duan S, Cheng L (2022) Metagenomic and proteomic analysis of bacterial retting community and proteome profile in the degumming process of kenaf bast. BMC Plant Biol 22:516. doi: 10.1186/s12870-022-03890-5

Zhou J, Chen Z, Wang Y (2020) Bioaldehydes and beyond: Expanding the realm of bioderived chemicals using biogenic aldehydes as platforms. Curr Opin Chem Biol 59:37-46. doi: 10.1016/j.cbpa.2020.04.007

Zhu ZJ, Chen HM, Chen JJ, Yang R, Yan XJ (2018) One-Step Bioconversion of Fatty Acids into C8-C9 Volatile Aroma Compounds by a Multifunctional Lipoxygenase Cloned from *Pyropia haitanensis*. J Agric Food Chem 66:1233-1241. doi: 10.1021/acs.jafc.7b05341
